# Impact of Vegetative Treatment Systems on Multiple Measures of Antibiotic Resistance in Agricultural Wastewater

**DOI:** 10.3390/ijerph15071295

**Published:** 2018-06-21

**Authors:** Lisa M. Durso, Daniel N. Miller, Christopher G. Henry

**Affiliations:** 1 United States Department of Agriculture (USDA), Agricultural Research Service (ARS), Agroecosystem Management Research Unit, 251 Filley Hall, UNL East Campus Lincoln, Lincoln, NE 68583, USA; dan.miller@ars.usda.gov; 2 Rice Research and Extension Center, University of Arkansas, Stuttgart, AR 72160, USA; cghenry@uark.edu; 3 Previously Biological Systems Engineering, University of Nebraska-Lincoln, Lincoln, NE 68583, USA

**Keywords:** antibiotic resistance, agriculture, wastewater, manure, cattle, antibiotic resistant bacteria, antibiotic resistance gene, tetracycline resistance gene, environment, vegetative treatment system

## Abstract

Wastewater is an important vector of antibiotic resistant bacteria and antibiotic resistance genes (ARB/G). While there is broad agreement that ARB/G from agricultural (ag) wastewaters can be transported through the environment and may contribute to untreatable infectious disease in humans and animals, there remain large knowledge gaps surrounding applied details on the types and amounts of ARB/G associated with different agricultural wastewater treatment options and different ag production systems. This study evaluates a vegetative treatment system (VTS) built to treat the wastewater from a beef cattle feedlot. Samples were collected for three years, and plated on multiple media types to enumerate tetracycline and cefotaxime-resistant bacteria. Enterobacteriaceae isolates (*n* = 822) were characterized for carriage of tetracycline resistance genes, and *E. coli* isolates (*n* = 673) were phenotyped to determine multi-drug resistance (MDR) profiles. Tetracycline resistance in feedlot runoff wastewater was 2-to-3 orders of magnitude higher compared to rainfall runoff from the VTS fields, indicating efficacy of the VTA for reducing ARB over time following wastewater application. Clear differences in MDR profiles were observed based on the specific media on which a sample was plated. This result highlights the importance of method, especially in the context of isolate-based surveillance and monitoring of ARB in agricultural wastewaters.

## 1. Introduction

Antibiotic resistance is a growing global health threat, with a projection of 10 million deaths per year attributable to previously-treatable infections by 2050 [1]. Although antibiotic resistant bacteria and antibiotic resistance genes (ARB/G) are found naturally in soils and water around the world [2,3,4,5], there is growing concern that input of large numbers of ARB/G into the environment via fecal wastes has adverse impacts for human, animal, and environmental health [3,4,6]. Sanitation and water quality are an important component of efforts to slow the spread of antibiotic resistant bacteria and antibiotic resistance genes (ARB/G) [7,8] and in the United States, public wastewater treatment plants have been shown to be effective for reducing ARB in the human waste stream [9]. 

In contrast to the standard primary, secondary, and tertiary treatments associated with human wastewater treatment, there is a great diversity of wastewater treatment options available in agricultural systems [10]. On-farm agricultural wastewaters include runoff from biosolids such as manure, litter, and compost, or liquid manure sludges and slurries [10]. The primary concerns surrounding agricultural wastewater are the release of nutrients and pathogens into surface waters [11,12]. In addition to these contaminants, there is growing awareness that manure-impacted wastewaters introduce additional antibiotic resistant bacteria and antibiotic resistance genes into the environment [4,10,13], and there is a growing concern that manure-associated ARB/G could indirectly impact human health, via transport in surface waters and soil [6,13,14]. 

Although there is great interest in the potential of ARB/G in food animal production to be transported through the environment and contribute to untreatable infectious disease in humans and animals, the data required to establish causal links, and for risk assessment in this area remain sparse [13,15,16]. There is a growing body of work characterizing and enumerating ARB/G in agricultural production settings [17,18,19,20,21,22,23,24,25], however multiple data gaps remain [16], including a specific need for information on number and type of ARB/G associated with land application of animal manures [13]. Complicating the efforts to inform environmental antibiotic risk assessment is the growing realization that specific answers depend, to a large extent, on the particular target that was measured [25,26], and the need to inform monitoring and surveillance efforts [8,15]. 

In order to provide information on ARGs associated with land application of agricultural wastewaters, this study examines ARB/G in a Vegetative Treatment System (VTS) used to treat beef cattle feedlot runoff [27]. A VTS works by collecting runoff, and then distributing it onto land with vegetation, where the plants will use the nutrients and water. It differs from a vegetative buffer strip in that a VTS is sized and graded specifically for the operation, and the release of the runoff is controlled. The relatively new design of VTS systems are seeing greater application in animal production operations, but their general function of repeated wastewater application to a defined treatment area could potentially enrich for ARB/G in their treatment areas. This study specifically investigates this concern.

## 2. Materials and Methods 

### 2.1. Site Description

This study was conducted on a central Nebraska vegetative treatment system (VTS) demonstration project, used to treat beef cattle feedlot runoff [27]. The initial feedlot capacity was slightly over 1000 head of feeder calves, and the feedlot owners were interested in expanding the operation to 1200 head while maintaining the same area footprint. A north lot was constructed to handle additional animals, and a pump-based VTS was installed to manage feedlot runoff, and prevent manure nutrients from contaminating nearby surface waters. 

**Figure 1:**
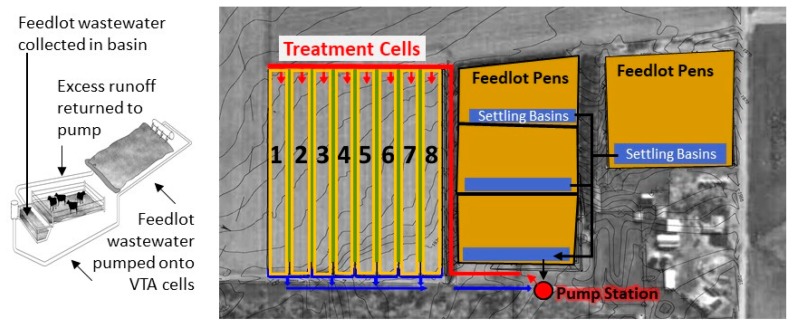
Vegetative Treatment System (VTS) design at beef cattle feedlot site. Feedlot runoff from pens is collected in settling basins, than routed to the pump station, and pumped onto treatment cells consisting of cool season grasses.

The VTS consisted of holding ponds designed to collect the runoff from a 25-year/24 h storm, a series of pipes and pumps that distributes runoff, and a set of eight vegetative fields to which the runoff is applied. The feedlot runoff was drained into a sump, conveyed uphill through 8-inch underground pipe to the eight vegetative treatment areas (VTAs), and applied at the top of the fields through irrigation pipe (Figure 1). The eight VTA cells at this site were built on Hord Silty Loam and Wann Fine Sandy Loam soil, planted to Meadow Brome, Tall Fescue, Orchard Grass, Smooth Brome, Intermediate Wheat, and Pubescent Wheat [27]. Experiments were started after the grasses had had three years to become established. Each VTA was 244 m × 19.5 m (4.5 ha), and they were separated by earthen berms. An additional berm was built along the bottom of the VTA to collect any excess runoff. Pipes channeled excess runoff back to the sump, creating a closed system. 

Following a rainfall event, the runoff from the feedlot pens was collected in unlined settling basins at the bottom of each pen. Cattle were excluded from the basins by fencing. After allowing solids to settle for 24–48 h, the feedlot wastewater was channeled into the pumping station, for distribution onto the VTAs. Rain that fell on the grassy VTA cells, but that did not infiltrate into the soil, was collected at the bottom of the VTA cells. After collection of the rainfall runoff, valves at the bottom of the VTA were opened, allowing the VTA rainwater runoff to also drain into the pumping station. 

### 2.2. Experimental Design

**Figure 2:**
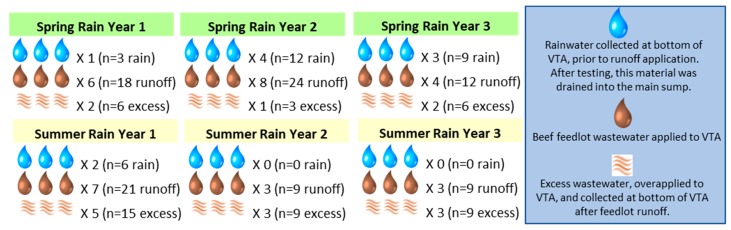
Experimental design.

In order to evaluate the efficacy of the VTS and the dynamics of runoff-associated antibiotic resistant bacteria and antibiotic resistance genes (ARB/G), samples were collected following individual spring and summer rainfall events, for three consecutive years, for a total of six collection time points (Figure 2). The original design included fall rainfall collection as well, but for the three years of the study, no fall rainfall events occurred that were large enough to require VTA application. Three types of samples were collected: rainfall runoff, feedlot runoff, and excess runoff. *Rainfall runoff* consisted of rain that had fallen on the VTA cells, but that did not infiltrate into the soil. VTA cells were paired (see Figure 1), and rainfall runoff was pooled for sets of two cells. The ARB/G in the VTA rainfall runoff represent organisms and genes that were still available for transport since the previous feedlot runoff application. *Feedlot wastewater* consisted of material that collected in the settling basins following a rain event. It included cattle manure and feedlot surface material particles suspended in the rainwater. *Excess wastewater* consisted of feedlot runoff that had been applied at the top of the VTA, and that was collected at the bottom of the VTA. Under normal operation, there was no excess wastewater. The VTA was usually managed so that only the amount of feedlot wastewater that could infiltrate the soil was applied to each VTA cell. For these experiments, feedlot wastewater was deliberately overapplied, so that we could evaluate the potential of the VTA as a mitigation for ARB/G. 

### 2.3. Sample Collection and Processing

All samples were collected in sterile 1 L screw-cap plastic Nalgene bottles, placed immediately in coolers for transport back to the laboratory, and processed the same day as collection. In the laboratory, samples were homogenized by shaking the bottles before aliquots were removed for analysis. Ten-fold dilutions were made using phosphate-buffered saline (ThermoFisher, Waltham MA, USA). In order to enumerate antibiotic resistant bacteria, and obtain isolates for multiple drug resistance testing commonly used in surveillance and monitoring programs [28], an Eddy Jet spiral plater (Neutec Group, Farmingdale, NY, USA) was used to plate all sample dilutions, in duplicate, onto MacConkey agar (MAC, Difco, Detroit, MI, USA), MacConkey with 16 μg mL^−1^ tetracycline at (TMAC), and MacConkey with 4 μg mL^−1^ cefotaxime (CMAC). Tetracycline was chosen because it is commonly used as a target to monitor antibiotic resistance in environmental samples, and cefotaxime was chosen because it is related to drugs that are fed to cattle and that are associated with drug resistant infections in children [29]. All plates were incubated overnight at 37 °C, and counted by hand 18–24 h after plating, using a standard spiral count procedure. Up to three isolates per sample were picked from each media type for further characterization, struck for isolation, and stored at −80 °C. All isolates were later grown overnight at 37 °C in in tryptic soy broth (Difco), and then confirmed as *Escherichia coli* using EC-MUG broth (Oxoid, Blasingstoke, Hampshire, UK). Isolates that did not display the fluorescence typical of *E. coli* were excluded from further analysis. 

Disk diffusion analysis was performed on all confirmed *E. coli* isolates against 12 drugs (*n* = 10,596 tests), according to Clinical Laboratory Standards Institute (CLSI) standard methods [30]. Briefly, Muller-Hinton broth (Beckton-Dickinson, Franklin Lakes, NJ, USA) was used to grow isolates overnight, cultures were adjusted to a standard optical density, and swabbed onto Mueller-Hinton agar plates. The CLSI clinical breakpoints were used to assign isolates to “resistant”, “intermediate”, or “sensitive” categories, although it is acknowledged that these isolates were of environmental origin, and were not confirmed human pathogens. For analysis, “intermediate” isolates were grouped with “sensitive” isolates, since both groups are not resistant. The term “sensitive” is used in results and discussion to include both the CLSI “sensitive” and CLSI “intermediate” isolates. The following drugs were used in the disk diffusion assays: amoxicillin/clavulanic acid (20 mg), ampicillin (10 mg), cefoxitin (30 mg), ceftriaxone (30 mg), chloramphenicol (30 mg), ciprofloxacin (5 mg), gentamycin (10 mg), kanamycin (30 mg), nalidixic acid (30 mg), streptomycin 10 mg, sulfamethoxazone trimethoprim (25 mg), and tetracycline (30 mg).

The tetracycline resistance gene profile of the isolates was probed using the polymerase chain reaction (PCR), following the protocols of Ng et al. [31] with Jumpstart RedTaq Master Mix (Sigma, St. Louis, MO, USA). Eleven targets were assayed, representing all three tetracycline resistance gene mechanisms. These included efflux targets *tet*(B), *tet*(C), *tet*(D), *tet*(K); *tet*(L), and *tetA*(P), *tet*(S); ribosomal protection targets *tet*(M), *tet*(O), *tet*(S), and the enzymatic target *tet*(X). Thermocycling conditions consisted of 1 cycle of 94 °C for 5 min; 35 cycles of 94 °C for 1 min, 55 °C for 1 min and 72 °C for 90 s; and one cycle of 72 °C for 5 min [31]. Positive control strains were created by cloning [26], and are available upon request. 

## 3. Results

A total of 178 wastewater and manure samples were collected over three years from the VTS following spring and summer rainfall events. Nebraska experienced drought over the course of the experiment, and no fall rainfall events were significant enough to allow for VTS operation. Also, there was no rainfall runoff available to be collected in the summers of year 2 and year 3. From the 178 samples, a total of 942 presumptive *E. coli* isolates were collected, and screened phenotypically and genotypically for selected antibiotic resistance targets (*n* = 444 from MAC, 467 from TMAC and 31 from CMAC).

### 3.1. Enumeration of Tetracycline and Cefotaxime Resistant Bacteria

Tetracycline resistant Gram negative enteric bacteria (bacteria that grew on MacConkey plates supplemented with 16 μg mL^−1^ tetracycline) were detected in all sample types (rainfall, wastewater, and excess) and at all collection times. Mean counts ranged between 1.12 and 5.12 log CFU/mL of sample (Table 1). Three-year mean values for rainfall runoff, feedlot wastewater, and excess wastewater were 1.56 log CFU/mL, 4.75 log CFU/mL, and 4.09 log CFU/mL, respectively (Table 2). 

### 3.2. Tetracycline Resistance Gene Assays

**Figure 3:**
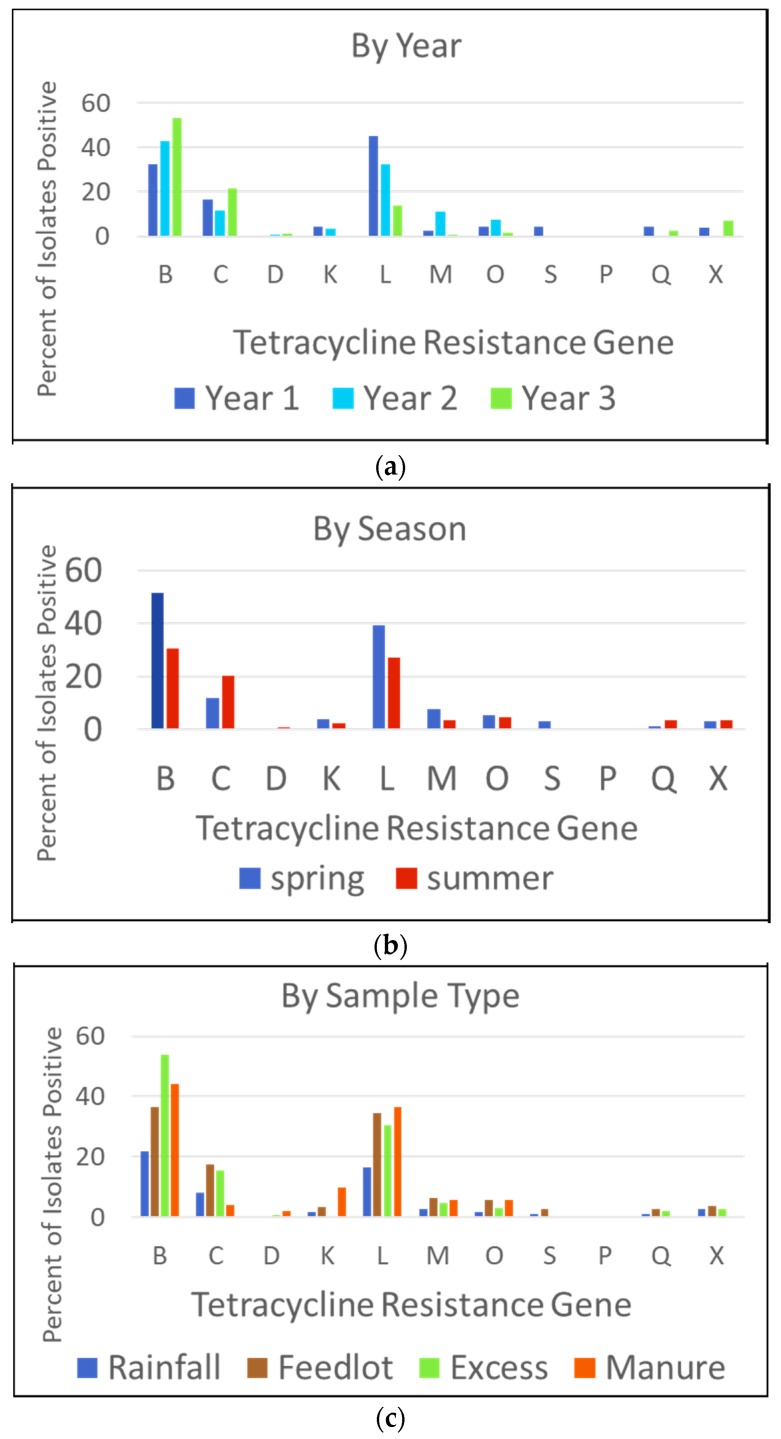
Prevalence of Tetracycline Resistance Genes. (**a**) By year (*n* = 822 isolates). (**b**) By Season (*n* = 822 isolates). (**c**) By sample type (*n* = 822 wastewater isolates and *n* = 55 manure isolates). Note that x axis letters refer to tetracycline resistance genes.

Up to three isolates were randomly selected per sample, for each of the three media types (MAC, TMAC, CMAC), streaked for isolation, and stored for antibiotic resistance gene (ARG) screening. Isolates were screened for the carriage of eleven tetracycline resistance genes, representing three tetracycline resistance mechanisms. Tetracycline resistance gene prevalence, by year, season, and sample type, is displayed in Figure 3. The prevalence of individual tetracycline resistance genes varied for all parameters observed. The *tet*(B) gene, was the most frequently detected gene in this sample set, followed by *tet*(L) and *tet*(C). Most targets (excluding *tet*(C) and *tet*(Q)) were more frequently detected in spring isolates, compared to summer isolates. When examining the results by sample type, individual ARGs were less frequently detected in the rainwater runoff samples than the feedlot wastewater and excess wastewater. 

### 3.3. Disk Diffusion Assays

**Figure 4:**
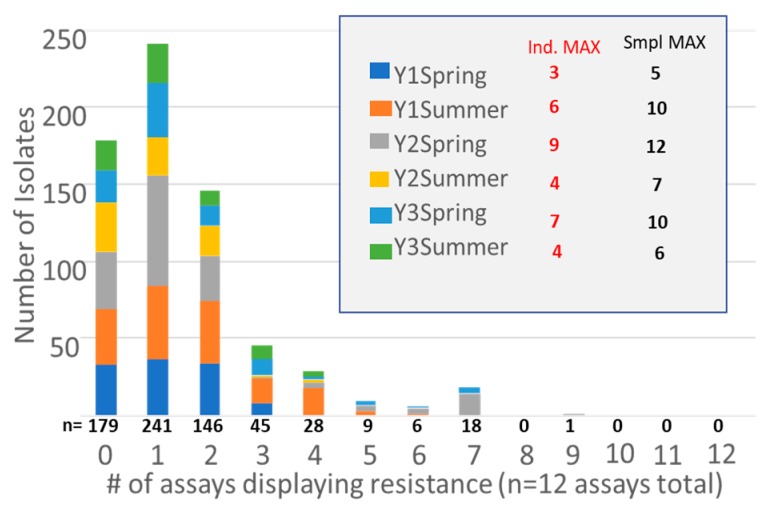
Number of multiple drug resistant (MDR) isolates, based on results of disk diffusion assays for 12 drugs. Bars display number of isolates with the listed number of resistances, 0–12. Total number positive across all sample times is listed directly under each bar. “Ind. MAX” indicates the maximum number of resistances in any one isolate from the given group. “Smpl MAX” indicates the total number of resistances positive for all isolates of the group (Y1Spring isolates displayed resistance to a total of five different drugs). Only samples that fluoresced in EC+MUG (confirmed as *E. coli*) were stamped for resistance patterns. Up to three isolates were picked per sample and media type.

Following *E. coli* confirmatory tests, 673 confirmed *E. coli* isolates were screened for multiple drug resistances using CLSI standardized disk diffusion assays. Of these, 27% (*n* = 179) were pan-susceptible to the 12-drug panel, 36% (*n* = 241) displayed phenotypic resistance to a single drug, 22% (*n* = 146) displayed phenotypic resistance to two drugs, and 7% displayed phenotypic resistance to three. Of the remaining 62 isolates, 18 displayed resistance to seven of the assay targets, and one isolate displayed resistance to nine drugs. The majority isolates displaying resistance to seven or more targets (*n* = 17/18) were picked from CMAC plates. The maximum number of resistances displayed by any one isolate at each of the six timepoints ranged from 3–9, and the maximum number of phenotypic resistances observed at any one time-point (all samples grouped together) ranged from 5–12 (Figure 4). MAC: *n* = 31, *n* = 293, and *n* = 120 for Rainfall Runoff, Feedlot Wastewater, and Excess Wastewater, respectively. For TMAC: *n* = 33, *n* = 293, and *n* = 141 for Rainfall Runoff, Feedlot Wastewater, and Excess Wastewater, respectively. For CMAC: *n* = 0, *n* = 26, and *n* = 5 for Rainfall Runoff, Feedlot Wastewater, and Excess Wastewater, respectively. Only samples that fluoresced in EC+MUG (confirmed as *E. coli*) were stamped for resistance patterns. Up to three isolates were picked per sample and media type.

**Figure 5:**
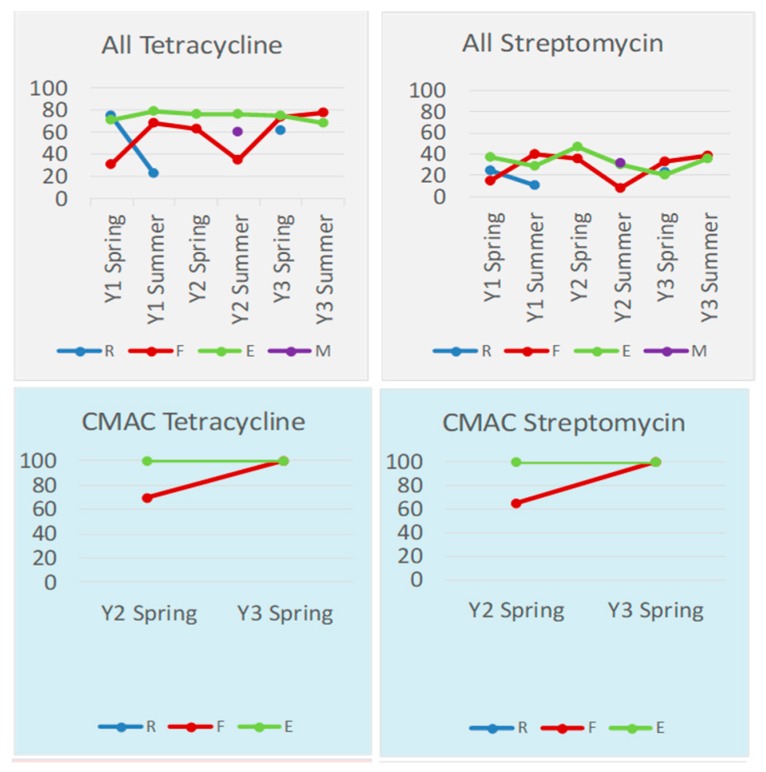

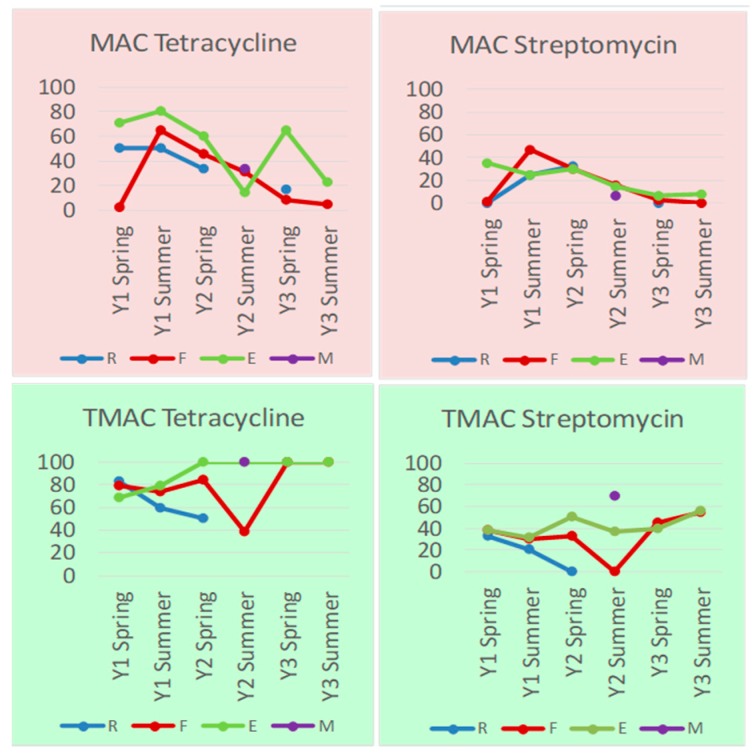
Percent of *E. coli* isolates phenotypically characterized as resistant to tetracycline and streptomycin, displayed by media from which they were initially isolated. MAC: *n* = 31, *n* = 293, and *n* = 120 for Rainfall Runoff (R), Feedlot Wastewater (F), and Excess Wastewater (E), respectively. For TMAC: *n* = 33, *n* = 293, and *n* = 141 for Rainfall Runoff, Feedlot Wastewater, and Excess Wastewater, respectively. For CMAC: *n* = 0, *n* = 26, and *n* = 5 for Rainfall Runoff, Feedlot Wastewater, and Excess Wastewater, respectively.

The relationships between the rainfall runoff, feedlot wastewater, and excess runoff for tetracycline and streptomycin are displayed in Figure 5. Tetracycline and streptomycin were the most frequently detected resistances in isolates. The results for the remaining targets are available in Supplementary Table S1 and Supplementary Figure S1 (Disk Diffusion Supp Data). The tetracycline results were typical of the majority of targets, with slightly fewer feedlot wastewater isolates, proportionately, displaying resistance to the drug, compared to excess wastewater. Rainfall runoff samples, when present, tended to have lower proportion displaying resistance for each target. 

## 4. Discussion

Mammalian feces are a rich source of bacteria, including antibiotic resistant bacteria and their genes (ARB/G). As such, wastewater treatment has an important role as a critical control point for remediation or mitigation. Due to the wide diversity of wastewater treatment options for food animal production systems, many qualitative data gaps remain before comprehensive risk assessment studies can be completed. This study provides seasonal and temporal data (3 years) on ARB/G in a VTS designed to manage wastewater from a beef cattle feedlot operation. Beef cattle are raised in every state of the U.S. [32], and there are an estimated 26,586 U.S. cattle feedlot operations. Data on ARB/G from beef production wastewaters is therefore an important component of efforts to understand and control ARB/G from agricultural production systems. 

In this study, tetracycline resistant bacteria (defined as the ability to grow on media with 16 μg mL^−1^ tetracycline) were cultured from all samples, with numbers from rainfall runoff generally 2–3 logs lower than feedlot wastewater. The rainfall runoff was a reflection of the persistence of bacteria and genes following application of wastewater to the soil, and the lower numbers indicate that the VTS was effective at reducing ARB over time. Manure-born bacteria thrive in the lower gastrointestinal tracts of mammals, where the temperatures are warm and stable. Following growth in the GIT, enteric bacteria are excreted into the environment where nutrients are often limiting, temperatures are generally cool and exceptionally variable, and competition from environmental bacteria is strong. While the bacteria can grow in the environment [33], most die before being re-inoculated into an animal host [34]. In light of these dynamics, and given the rich bacterial communities of soil, plants, and the rhizosphere, it is not surprising to see the observed lower numbers of ARB in the rainfall runoff samples. Compared to tetracycline, cefotaxime resistance (growth on media containing 4 μg mL^−1^ cefotaxime) was very low (less than 200 CFU per/mL) and was only detected during spring events in years two and three. An increase was noted between years two and three, but due to the small number of measurements, it is difficult to know if this is a long-term trend. Whether this is true seasonality is interesting to consider, but more study is needed to see if this is a consistent pattern in wastewater. It should be noted that no cefotaxime resistant microorganisms were detected in the rainfall runoff. 

Similar to the ARB results, tetracycline ARGs were consistently present in lower numbers in the rainfall runoff, compared to the wastewater samples. Unlike phenotypic resistance, which is characterized by a single measure (growth in the presence of a defined concentration of tetracycline), genotypically there are dozens of genes that code for tetracycline resistance [35,36]. Here we examined presence/absence of eleven genes, representing all three tetracycline resistance mechanisms. The most informative tetracycline resistance gene targets for this sample set were *tet*(B) and *tet*(L) (Figure 3), both coding for efflux proteins. When examined by season, both *tet*(B) and *tet*(L) occurred in a higher proportion of the spring isolates compared to the summer isolates. A third target, *tet*(C) was present in over 10% of isolates, but was isolated from a higher proportion of summer isolates. These particular results reinforce the idea that individual ARGs each have their own ecology [25,26], and the outcomes of data collection can vary depending on which target is chosen as a measure of resistance. In native Nebraska prairie soils, *tet*(L) and *tet*(B) were also informative, along with *tet*(D) and *tet*(O), which were detected in 54 and 38 percent of the samples, respectively. In the current study, *tet*(D) was only found in four isolates, and *tet*(O) was found in 38/822 isolates, with most of the *tet*(O) isolates (*n* = 30) coming from the feedlot runoff samples [26]. In contrast, the most frequently detected tetracyline resistance genes in Nebraska organic farm soils were *tet*(Q), *tet*(S) and *tet*(X) [25]. Examining results from all three sites as a way to determine which tetracycline resistance genes might be the most relevant for monitoring and screening, it is clear that the heterogeneity in tetracycline resistance gene prevalence makes choice of a single target or set of targets difficult, without prior knowledge of the tetracycline resistance gene profile of the source materials. Of note, the prairie and organic farm studies surveyed whole community samples, while the current study examined isolates. As with Microbial Source Tracking, protocols will need to start by specifically defining the research question, and include identification of the target, identification of the method (gene based or culture-based), and identification of the analytical approach [37]. As with microbial source tracking, baseline information of sites and targets will improve study outcomes 

In addition to plating and ARG screening, *E. coli* VTS isolates were characterized by their antibiotic resistance patterns to better understand the diversity and distribution of resistance in the microbial community. The majority of isolates were considered clinically sensitive to the antibiotics we used for screening. Both single and multiple resistant *E. coli* strains were more likely to be found in the feedlot wastewater and excess wastewater, compared to rainfall runoff (Figure 3). The rainfall runoff provides information on the functioning of the VTS over time, since they provide insight into what organisms survive on and are released from the VTS after repeated feedlot wastewater applications. The lower numbers of ARGs found in rainfall runoff suggests that the VTA is effective in remediating the ARGs from feedlot runoff, over time. As might be expected, isolates picked off of plates containing antibiotics were more likely to display multiple resistances, likely due to the selective nature of the isolation, demonstrating the impact of isolation method on interpretation of results. Isolates from the TMAC plates were more likely to display clinical resistant to at least one antibiotic in our panel (94% of TMAC isolates categorized as resistant based on CLSI disk diffusion results), compared to isolates picked off of the plain MAC plate (45% resistant). Of interest is that isolates from the CMAC plates were frequently found to be resistant to multiple drugs, with the majority of isolates (66%) displaying resistance to 5 of the 11 drugs used for screening in this study. So although the prevalence of the cefotaxime-resistant bacteria is low compared to the tetracycline resistance phenotype, their high degree of multi-drug resistance means that they are an important component to monitor in these systems. Isolate resistance expressed in terms of years or seasons show no distinct patterns other than the aforementioned detection of cefotaxime resistant microorganisms only during early spring.

VTS have been developed and built as an alternative to conventional full containment systems (holding pond systems) for managing manure, litter, and/or process wastewater discharges from animal feeding operations. VTS are used most often by small and medium-sized feedlots (less than 1000 head) [38]. An estimated 93% of feedlot operations fit this category (NCBA). In this study a VTS was constructed to serve an operation with greater than 1000 animals. Already in its third year of operation, we noticed that the infiltration capacity of the soils increased each year. In general, land application of manure-impacted solids and liquids results in an initial spike in target levels [39], followed by a decline over time [20,40,41]. We observed similar trends in this beef cattle feedlot VTS. The VTS evaluated as part of this study was effective at preventing the release of ARB/G from agricultural wastewaters into nearby surface waters, and successful at reducing ARB/G concentrations over time.

## 5. Conclusions

In this study, we evaluated tetracycline and cefotaxim resistance in bacteria and genes from a demonstration VTS, including feedlot wastewater, and background resistance from rainfall on application fields. Tetracycline resistance in the wastewater was 2 to 3 orders of magnitude higher, compared to rainfall runoff from the vegetated fields, indicating decay of ARB/G over time on the VTA. 

In addition to collecting data to inform risk models and better understand the ecology of tetracycline and cefotaxime resistance in agricultural wastewaters, our data provide an insight into the influence of microbiological methods on the detection of ARB/G in agricultural systems. Our data clearly reveal that the conclusions one can draw from both culture-based and PCR-based methods depends on the targets measured and the methods used. In this study for instance, the perception of “how much” tetracycline resistance was present in the system would vary greatly depending on which specific tetracycline resistance gene was chosen. It has been suggested that *tet*(M) be used for monitoring resistance in environmental samples [42]. In this instance, *tet*(M) prevalence in our *E. coli* isolates was less than 10%, compared to *tet*(B), which was 30–50%, depending on the year. And the amount of multiple-drug-resistance observed in *E. coli* isolates was strongly impacted by the type of media on which the sample was first plated. This media-based difference has important implications for antibiotic resistance monitoring and surveillance efforts, particularly in the context of wastewater and public health. It will be essential to standardize not only which targets are measured, but also how the isolates are first cultured from the environment. Based on results from this study, if detection of low level target is desired, use of selective media may prove useful.

## Figures and Tables

**Table 1:** Abundance of antibiotic resistant * enteric microorganisms in wastewater samples collected from the vegetative treatment system.

Event/Date	Water Source ^†^	Number of Samples	Tetracycline Resistant G^−^ Enteric Bacteria		Cefotaxime Resistant G^−^ Enteric Bacteria
			log CFU/mL	SD	Range CFU/mL
**Year 1**	Rainwater Runoff	3	1.75	0.05	None Detected
**Spring**	Feedlot Wastewater	18	5.12	0.21	None Detected
	Excess Wastewater	6	5.20	0.14	None Detected
**Year 1**	Rainwater Runoff	6	1.87	0.57	None Detected
**Summer**	Feedlot Wastewater	21	4.65	0.23	None Detected
	Excess Wastewater	11	4.82	0.36	None Detected
**Year 2**	Rainwater Runoff	12	1.12	0.16	None Detected
**Spring**	Feedlot Wastewater	24	5.08	0.18	0 to 180
	Excess Wastewater	3	4.95	0.04	40 to 160
**Year 2**	Rainwater Runoff	--- ^‡^	---	---	---
**Summer**	Feedlot Wastewater	9	3.11	0.39	None Detected
	Excess Wastewater	9	2.65	0.33	None Detected
**Year 3**	Rainwater Runoff	9	1.50	0.39	None Detected
**Spring**	Feedlot Wastewater	12	3.54	0.22	0 to 20
	Excess Wastewater	6	3.65	0.27	0 to 20
**Year 3**	Rainwater Runoff	---	---	---	---
**Summer**	Feedlot Wastewater	10	3.41	0.35	None Detected
	Excess Wastewater	9	3.59	0.31	None Detected

* Resistance defined for the purpose of this study as growth in the presence of 16 μg mL^−1^ tetracycline, or 4 μg mL^−1^ cefotaxime. ^†^ Rainwater Runoff is rainfall runoff from the treatment cells prior to wastewater application; Feedlot Wastewater is runoff collected from the surface of beef cattle feedlot pens and applied to the treatment cells on the day of sampling; Excess Wastewater is wastewater that has not infiltrated during application and runoff of the treatment area. ^‡^ no rainwater runoff available for collection.

**Table 2:** Annual average abundance of tetracycline resistant bacteria in VTS.

	Rainfall Runoff *		Feedlot Wastewater *		Excess Wastewater *	
Period	Log CFU/mL	SD	Log CFU/mL	SD	Log CFU/mL	SD
2010	1.83 ^a^	0.45	4.87 ^a^	0.32	4.93 ^a^	0.35
2011	1.12 ^b^	0.16	4.54 ^b^	0.92	3.22 ^b^	1.08
2012	1.50 ^ab^	0.39	3.48 ^c^	0.28	3.61 ^b^	0.28
3 Year Mean	1.56	0.48	4.43	0.81	4.09	0.96
Spring	1.41 ^a^	0.35	4.75 ^a^	0.68	4.53 ^a^	0.77
Summer	1.87 ^b^	0.57	3.99 ^b^	0.77	3.89 ^b^	0.99
Fall	---	---	---	---	---	---

* Rainfall Runoff (*n* = 9, *n* = 12, and *n* = 9 for Year 1, 2, and 3, respectively), Feedlot wastewater (*n* = 39, *n* = 33, and *n* = 22 for Year 1, 2, and 3, respectively), and Excess Wastewater (*n* = 21, *n* = 12, and *n* = 15 for Year 1, 2, and 3, respectively). ^abc^ Values with different superscripts within a group (year or season) differ (*p* < 0.05) as determined a Student’s *t* test statistic.

## Supplementary Materials

The following are available online at http://www.mdpi.com/1660-4601/15/7/1295/s1, Figure S1: Percent of *E. coli* isolates displaying phenotypic resistance (*n* = 12 targets assayed); Table S1: Disk Diffusion results displayed by target and time.
